# Abstract of the Proceedings of the Buffalo Medical Association

**Published:** 1878-11

**Authors:** Joseph Fowler

**Affiliations:** Secretary


*ART. II.—Abstract of the Proceedings of the Buffalo Medical Association.* Reported by JOSEPH FOWLER, M. D., *Secretary,* October 1, 1878.

Meeting called to order by the President, Dr. Lothrop. Members in attendance were: Drs. Lothrop, Rochester, Cronyn, Wyckoff, Moody, Hauenstein, Brecht, Howe, Wetmore and Fowler, and by invitation, Dr. Nichols.

Proceedings of August meeting read and approved.

Dr. Rochester reported the following case, and presented the pathological specimen for inspection:

Early in the year 1875, Michael Hayes, aged 28 years, called at his office to consult him concerning an abscess which had recently appeared in the right iliac region. It was painful, circumscribed, hard and accompanied with considerable febrile disturbance. The possibility of it being a hernia or an enlarged gland was excluded. It presented all the appearances of a perityphilitic abscess, and, in his judgment, a proper one for operation. While the patient was in this condition, he invited Dr. J. F. Miner to see him in consultation and to go prepared to operate. When they visited the patient it was found that the painful tumor, so apparent the day before, had entirely disappeared, followed by almost complete cessation of pain. It first occurred to him that there might have been an error in diagnosis, but the patient at once informed him that he was passing fœcal matter through his penis, and did so in the presence of both physicians. He remained under obserservation for about six months, after which he was lost sight of. Dr. Rochester subsequently was informed that he had gone to Bellevue Hospital to be operated upon, but receiving no encouragement returned to Buffalo, and came under the care of Dr. Boardman, of this city. He recently died, and Dr. B. invited him to be present at the *post mortem.* The subject was greatly emaciated, there was no swelling of any part of the abdomen. In the right iliac region the intestines were firmly adherent to each other and adjacent parts. It was difficult to isolate the caput coli. The appendix was firmly attached to the abdominal wall. The prostate gland was considerably hypertrophied, the rectum was on the right side, beneath the abscess and bound to surrounding parts. Upon cutting down into the situation of the abscess, a sack was found containing pus and fœcal matter. There was a communication between it and the appendix vermiformis; also between the abscess and the rectum, and still another into the bladder, and a fourth into the head of the colon, but none into the peritoneal cavity.

The specimen as presented by Dr. Rochester showed portion of the bladder, colon, rectum and appendix in their normal relations to each other. The removal of these parts was accomplished under great difficulties, and he desired to thank Dr. Boardman for the specimen, and the care taken to secure it.

This completes twenty three cases coming under Dr. R.'s observation, six of which recovered—two by spontaneous evacuation, one by operation, and of three it was difficult to decide how. Think an early operation in the case reported might have saved the life of the patient.

Dr. Cronyn said he had seen the man after his return from New York, and his history, as given by himself, agreed with what Dr. Rochester had said. Saw him soon after his return, when he was complaining of colic and had a swelling in the right iliac region, and passing pus through the urethra. It was his opinion that an abscess had formed about the caput coli and opened into the bladder or urethra. He thought it a very curious circumstance, and upon looking up the literature of the subject found only three similar cases reported, all of which were fatal. The propriety of operating for restoration of the fistulous opening was discussed. Thinks it possible that if the abscess had been opened the result might have been otherwise; would recommend operating early, especially as the cases prove fatal in time.

Dr. R. remarked: In some cases it would be madness to operate; when the abscess occurs from external violence there would be greater assurance of success. The Doctor recalled to mind a boy 10 years old, who had been kicked in the right iliac region by another boy, resulting in an abscess in that situation. Death ensued, and it was found that sloughing of the appendix had taken place. He recollected of reading a report of a similar case many years ago which occurred in England.

Under the head of "prevailing diseases," Dr. Rochester said he believed there were less autumnal disorders prevailing than usual, but an unusual amount of fever called malarial, typhoid and typho-malarial, according to the symptoms presented. In some cases there was a great variation of temperature in the same day. Some of the cases had diarrhœa or epistaxis. The abdomen was not tympanitic, and the tongue did not present the usual appearance characteristic of typhoid fever. Did not think he was warranted in calling the disease typhoid. This form of disease had proved very obstinate in some cases, lasting about three weeks, and liable to relapse, often taking on the form of intermittent fever of a low type. In one case cerebro spinal meningitis was the most alarming symptom. Had seen, also, a number of cases of scarlet fever.

Dr. Wetmore believed there was more miasmatic fever in the city than ever before. Had four cases which he called typho-malarial. There was tenderness of the abdomen and petechial spots. The temperature was variable. One case had a relapse. First treated the disease by giving four grains of quinine every four hours, with small doses of strychnia and arsenic; gave 20 to 30 grains of quinine each day for four days to a child twelve years old, but could not say that it abbreviated the disease.

Dr. Hauenstein said he had seen several cases; one had well marked typhoid symptoms. Epistaxis was profuse, the temperature 102½ to 104½° F. There was no tympanitis, tenderness or diarrhœa. Another case, a girl had very severe menorrhagia with the other symptoms.

Dr. Johnson said he had observed some cases quite similar to those just described. Had seen one case of typhoid fever, and a few cases of scarlet fever which assumed an intermittent form.

Dr. Wyckoff reported having treated a number of cases of fever of the same nature described, one of which terminated fatally. She was very ill for three or four weeks; had delirium, a tympanitic abdomen and high temperature. She improved for a time and then grew worse. At the end of six weeks it seemed as if she would recover, when suddenly vomiting set in and she died in eight hours. Two other cases had phlebetis as a complication. The temperature in all his cases was high and variable, often higher in the morning than in the evening, reaching 104°, F.

Another case had congestion of the lungs and bloody expectoration, was delirious, had epistaxis and temporary loss of voice and hearing.

Dr. Moody said she had seen one case with a temperature of 104°, which improved under the use of four grains of quinine every three hours.

Dr. Cronyn had seen a number of such cases, and was glad to hear Dr. Rochester say the disease was not typhoid fever. Many of them have diarrhœa, but not so obstinate as in typhoid fever. There was a tendency to vomiting, and tenderness over the abdomen, most susceptible near the umbilicus, but no petechial spots. Remission and exacerbation of fever in the same day were remarkable. Think quinine of little value when administered beyond its power to effect a reduction of temperature; he gave most of his patients hydrochloric acid, perhaps a little quinine and dovers powder. Was disposed to give but little medicine, but was careful of nutrition. The delirium was of rather a pleasant nature; his patients were not furious, but saw pleasant sights. There was distention of abdomen in only one case, and a tendency to relapse in all. Did not think typho-malarial a good name to apply to the disease. Thought perhaps the water might have something to do with its production. Three servants who had been drinking from the same well came to his office all presenting like symptoms of typhoid fever.

Dr. Rochester said he attended recently a patient in the eastern part of the city who was suffering from irritation of the bowels and fever, and was informed that there were about forty or fifty persons in the same neighborhood similarly affected. He saw some of the water from the well these people drank, and it was very clear and cool.

Dr. Howe said as the discussion had turned upon our water-supply, he might say that at a recent meeting of the Microscopic Society crystals of uric acid were shown to be in the water of a well, located in the eastern part of the city, and in that of another on Maryland street there were found woolen fiber. This was suggestive as it might be the remains of the bag used to drown the cats.

The application of Dr. Cary was presented and took the usual course.

The Association then adjourned.

